# Differential Oxygen Exposure Modulates Mesenchymal Stem Cell Metabolism and Proliferation through mTOR Signaling

**DOI:** 10.3390/ijms23073749

**Published:** 2022-03-29

**Authors:** Inês Moniz, João Ramalho-Santos, Ana F. Branco

**Affiliations:** 1 CNC—Centre for Neuroscience and Cell Biology, CIBB—Centre for Innovative Biomedicine and Biotechnology, University of Coimbra, Azinhaga de Santa Comba, Polo 3, 3000-548 Coimbra, Portugal; inescalasimo@hotmail.com; 2 Department of Life Sciences, University of Coimbra, Calçada Martim de Freitas, 3000-456 Coimbra, Portugal

**Keywords:** mesenchymal stem cells, hypoxia, metabolism, mTOR, cobalt chloride

## Abstract

Mesenchymal stem cells reside under precise hypoxic conditions that are paramount in determining cell fate and behavior (metabolism, proliferation, differentiation, etc.). In this work, we show that different oxygen tensions promote a distinct proliferative response and affect the biosynthetic demand and global metabolic profile of umbilical cord-mesenchymal stem cells (UC-MSCs). Using both gas-based strategies and CoCl_2_ as a substitute for the costly hypoxic chambers, we found that specific oxygen tensions influence the fate of UC-MSCs differently. While 5% O_2_ potentiates proliferation, stimulates biosynthetic pathways, and promotes a global hypermetabolic profile, exposure to <1% O_2_ contributes to a quiescent-like cell state that relies heavily on anaerobic glycolysis. We show that using CoCl_2_ as a hypoxia substitute of moderate hypoxia has distinct metabolic effects, when compared with gas-based strategies. The present study also highlights that, while severe hypoxia regulates global translation via mTORC1 modulation, its effects on survival-related mechanisms are mainly modulated through mTORC2. Therefore, the experimental conditions used in this study establish a robust and reliable hypoxia model for UC-MSCs, providing relevant insights into how stem cells are influenced by their physiological environment, and how different strategies of modulating hypoxia may influence experimental outcomes.

## 1. Introduction

Mesenchymal stem cells (MSCs) have been consistently reported as promising candidates in functional tissue engineering and regenerative therapies [1,2,3]. The umbilical cord comprises a specific stem cell niche where precise oxygen (O_2_) levels are paramount to maintain and optimize stem cell function. In this tissue, umbilical cord-mesenchymal stem cells (UC-MSCs) reside under hypoxic conditions, ranging from less than 1% O_2_ (severe hypoxia) to 5% O_2_ (moderate hypoxia) [4].

The hypoxia-inducible factor 1 (HIF-1) complex has emerged as one of the main components of hypoxia response in most cell lines [5,6]. One of its isomers, HIF-1α, is a transcriptional factor regulated by O_2_ and mobilized by hypoxia, with the crucial role of suppressing mitochondrial respiration while increasing glycolytic enzyme expression [6]. Given that MSCs reside under these hypoxic physiological settings, in vitro culture under atmospheric oxygen concentrations (21% O_2_, normoxia) can be detrimental to the therapeutic value of MSCs, by hindering their plasticity, proliferation, and clonogenic capacity [7].

In recent years, hypoxia preconditioning has been acknowledged as an adequate priming technique to both enhance and preserve MSC bioactivity and biological identity, respectively [8]. Nevertheless, studies on hypoxia preconditioning predominantly show the effects of moderate hypoxia on MSC differentiation and expansion—with several authors reporting an increase in proliferation following hypoxic treatment [9,10,11]. However, severe hypoxia may have underlying advantages and might be key to promoting a hypo-proliferative state that safeguards the cell and guarantees its longevity. In fact, numerous organisms and cell lines temporarily cease cellular activity as a protective mechanism against adverse environmental conditions (e.g., nutrient depletion) [12,13]. This transitory arrest allows the cell to escape cellular senescence, DNA damage and oxidative stress associated with oxidative phosphorylation (OXPHOS) [14], and this preconditioning technique might be beneficial for the long-term survival of MSCs.

Quiescence is frequently associated with a suppression in global protein synthesis, metabolic activity, and energetic demand [14]. In this regard, the mammalian target of rapamycin (mTOR) pathway plays a key role acting as a sensor and integrator of a large variety of environmental cues (e.g., growth factors, nutrients, and O_2_ tension) that are linked to cell cycle arrest, metabolic quiescence, and biosynthetic requirements [15]. Moreover, the pharmacological inhibition of both mTOR complexes (mTORC1 and mTORC2) with INK-128 has been proven to induce a reversible “paused-like” state in mouse embryonic stem cells (mESCs) and bone marrow MSCs (BM-MSCs) [16,17,18,19].

While hypoxic settings are primarily induced by decreasing O_2_ concentrations, some chemical hypoxia-mimetic agents (e.g., cobalt chloride) have been indiscriminately used as a substitute for physical hypoxia, despite the still elusive effects prompted by this alternate method [20]. The use of CoCl_2_ also allows us to distinguish the hypoxic effects produced specifically by HIF-1α-related pathways.

Based on these theoretical considerations, in the present study, we aimed to perform a comparative characterization of different hypoxic settings on the behavior of UC-MSCs, focusing on proliferation and metabolism. We also aimed to determine whether severe hypoxia, either physical or chemical, could prompt a mTOR-dependent quiescent state in UC-MSCs.

## 2. Results

### 2.1. Severe Hypoxia Promotes a Quiescence-Like State

**Figure 1:**
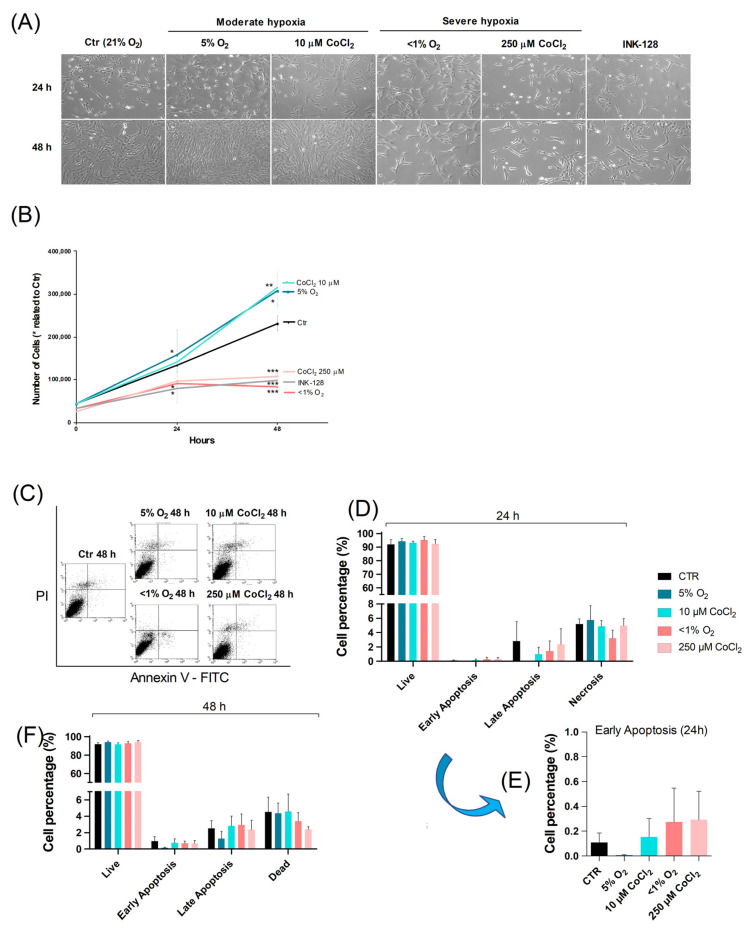
Hypoxic stimuli have opposing effects on UC-MSC proliferation. UC-MSCs were cultured under different O_2_ and CoCl_2_ levels—control (21% O_2_), and moderate (5% O_2_ and 10 μM CoCl_2_) and severe (<1% O_2_ and 250 μM CoCl_2_) hypoxia—and cultured with INK-128, for 24 and 48 h. (**A**) Representative images of plated UC-MSCs treated under control and experimental conditions, captured in a phase-contrast microscope (10× magnification). (**B**) Total number of live cells counted after 24 and 48 h incubation period, under control and experimental conditions, from a minimum of 3 independent experiments. Results are represented as means with SEM. Statistical significance considered when * *p* < 0.05%, ** *p* < 0.01 and *** *p* < 0.001. (**C**) Representative flow cytometry dot plot and the respective 24 h (**D**) and 48 h (**F**) quantification of live, early apoptotic, late apoptotic, and necrotic populations through Annexin V/PI staining. (**E**) Enhanced scale of the early apoptosis bars of graph D.

To determine the effects of different O_2_ levels on UC-MSC proliferation, cells were exposed to moderate (5% O_2_) and severe (<1% O_2_) hypoxia for 24 and 48 h Likewise, low (10 μM) and high (250 μM) concentrations of CoCl_2_ were used for the same time periods. To induce an mTOR-dependent arrested proliferative state for comparative purposes, cells were also cultured under 100 nM of the mTOR inhibitor INK-128. At all time points, the control and experimental groups were closely monitored under a phase-contrast microscope (Figure 1A). To quantify and evaluate the effects of different oxygenation settings on proliferation, a growth curve was also generated (Figure 1B).

Differences between conditions were more evident after 48 h. UC-MSCs incubated under 5% O_2_ and treated with 10 μM CoCl_2_ exhibited similar proliferative profiles—with increased proliferative rates and decreased doubling times. However, cells that underwent severe hypoxia and 250 μM CoCl_2_ revealed a decrease in proliferation, on par with cells incubated with INK-128. Furthermore, cells exposed to these experimental conditions also maintained their 24 h cell count, suggesting that severe hypoxia and high concentrations of CoCl_2_ could be causing an arrested, quiescent-like state in UC-MSCs.

Cell death was also factored in, to ensure that an apparent decrease in live-cell count was a by-product of quiescence rather than cell death. The total number of dead cells was counted by trypan blue staining (data not shown), while the percentage of live, apoptotic, and necrotic cells was monitored using flow-cytometry (Figure 1C–F). Neither physical nor chemical hypoxia significantly increased the percentage of apoptotic/necrotic cells.

### 2.2. Oxidative Phosphorylation Shifts toward Glycolysis in Severe Hypoxia

Different proliferative profiles can have distinct metabolic requirements. A quiescent state is characterized by a decrease in metabolic activity and energetic demand [21]. Glycolysis and oxidative phosphorylation are the two major energy-producing pathways in the cell, and metabolic transitions between these two pathways take place as an adaptation to environmental changes.

**Figure 2:**
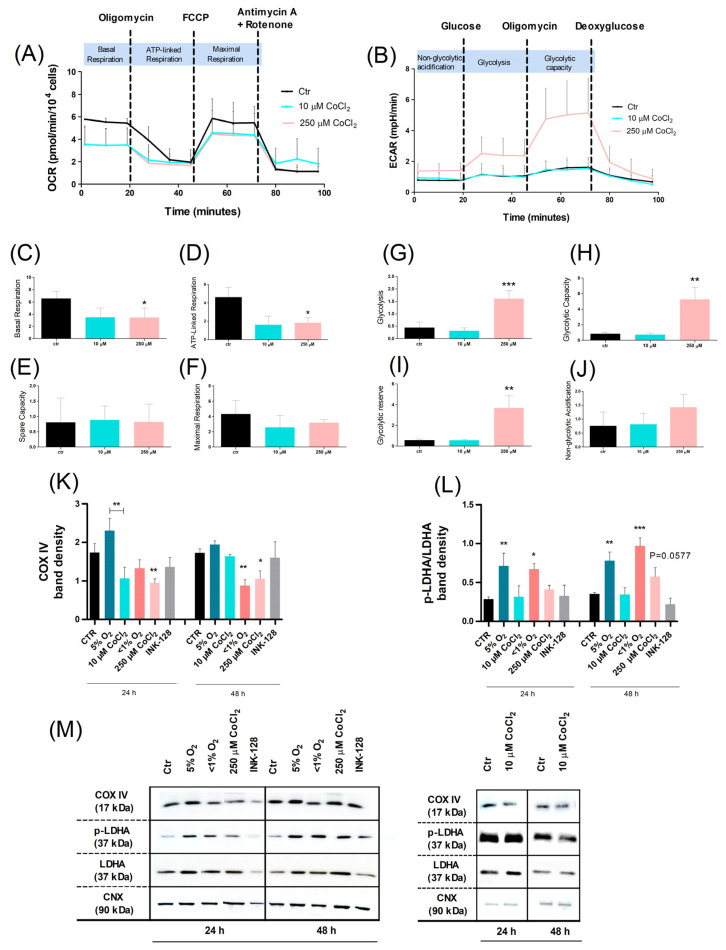
Comparative analysis of the effects of hypoxia and CoCl_2_ on UC-MSC metabolism. UC-MSCs were cultured for 24 and 48 h under different O_2_ levels (21%, 5%, <1%) or CoCl_2_ concentrations (10 μM and 250 μM); cells were treated with the mTOR dual inhibitor INK-128 as a positive control for a quiescent state. To assess both the oxidative metabolism and the extracellular acidification rate of UC-MSC, OCR and ECAR were measured, respectively, with a Seahorse XF24 Analyzer. Three measures were performed after each drug injection. (**A**,**B**) Schematic image of the metabolic modulator injection sequence and the parameters that could be obtained with the OCR and ECAR assays. (**A**) OCR profile throughout the experiment, (**C**) basal respiration, (**D**) ATP-linked respiration, (**E**) spare capacity, and (**F**) oxygen maximal consumption. (**B**) ECAR profile throughout the experiment, (**G**) glycolysis, (**H**) glycolytic capacity, (**I**) glycolytic reserve, and (**J**) non-glycolytic acidification. Spare capacity (OCR) and glycolytic reserve (ECAR), not represented in the image, were calculated using the formulas (maximal respiration—ATP-linked respiration) and (glycolytic capacity—glycolysis), respectively. Protein level analysis of (**K**) COX IV and (**L**) p-LDHA/LDHA and the (**M**) respective representative images, performed through Western blot. The values were normalized by the expression of the loading control calnexin (CNX). The values are represented as means with SEM of at least three independent experiments. Statistical significance considered when * *p* < 0.05%, ** *p* < 0.01, and *** *p* < 0.001.

As such, and since oxygen availability influences global metabolic dynamics, a live-cell metabolic assay was carried out, using a Seahorse XF Analyzer (Agilent Technologies, CA, USA). Accordingly, the oxygen consumption rate (OCR) and extracellular acidification rate (ECAR) were monitored (Figure 2A,B). As this assay required an overnight incubation in the experimental settings, the assay was not applicable to physical hypoxia-treated cells.

OCR evaluation confirmed a decrease in mitochondrial function after exposure to both concentrations of CoCl_2_ with a significant decrease in basal respiration and ATP-linked respiration from cells treated with 250 µM CoCl_2_ (Figure 2C,D). These altered parameters allowed us to identify the endogenous ATP demand of the cell and estimate the respiration that was used to drive mitochondrial ATP synthesis, respectively. ECAR levels were also increased for cells treated with 250 µM CoCl_2_ (but not 10 µM), which translated into an increase in glycolytic function, capacity, and reserve (Figure 2G–I).

For a more detailed metabolic characterization, the levels of key metabolic regulatory proteins were also assessed (Figure 2K,L). In agreement with the previous results, protein expression analysis revealed that the expression of the mitochondrial electron transport chain terminal enzyme, COX IV, also differed according to the severity of the hypoxic condition. It was found that <1% O_2_ and 250 µM CoCl_2_ (hypo-proliferating cells) decreased COX IV expression levels (48 h). In contrast, 5% O_2_ and 10 µM CoCl_2_ (hyper-proliferating cells) exhibited no significant changes in relation to the control (48 h); notwithstanding, cells exposed to the latter conditions were significantly distinct: 10 µM CoCl_2_ decreased COX IV expression levels, whereas 5% O_2_ promoted the expression of this enzyme.

Interestingly, the results also showed an increase in the glycolytic enzyme lactate dehydrogenase LDHA function (inferred by the p-LDHA/LDHA ratio), in cells exposed to physical hypoxia (5% O_2_ and <1% O_2_) as opposed to cells treated with their respective CoCl_2_ counterparts, thus emphasizing a significant metabolic difference between physical and chemical hypoxia. It is worth noting that there was a statistically increasing trend in LDHA expression in cells treated with 250 µM CoCl_2_ (*p* = 0.0577), which was consistent with the ECAR evaluation.

Taken together, our results revealed that under severe hypoxic conditions (<1% O_2_ and 250 μM CoCl_2_), UC-MSC metabolism relied heavily on anaerobic glycolysis, rather than oxidative phosphorylation, which helped the cells to maintain a quiescent state. On the other hand, UC-MSCs exposed to moderate physical hypoxia (5% O_2_) exhibited an overall hyperproliferative and hypermetabolic state that relied on both oxidative phosphorylation and glycolysis; conversely, incubation with a moderate chemical hypoxia mimetic (10 μM CoCl_2_) provoked an overall hypometabolic state.

### 2.3. Severe Hypoxia Influences mTOR Signaling

Quiescence is frequently associated with a suppression in global protein synthesis. The dual mTOR inhibitor, INK-128, induces a quiescent state in several stem cell cultures by downregulating the active form of its effectors; such effectors include the eIF4E-binding protein 1 (4EBP1) and the ribosomal protein S6 kinase beta-1 (S6K1), which are involved in the overall biosynthesis process [15]. Notably, mTOR is also known to be sensitive to different oxygen tensions [22].

**Figure 3:**
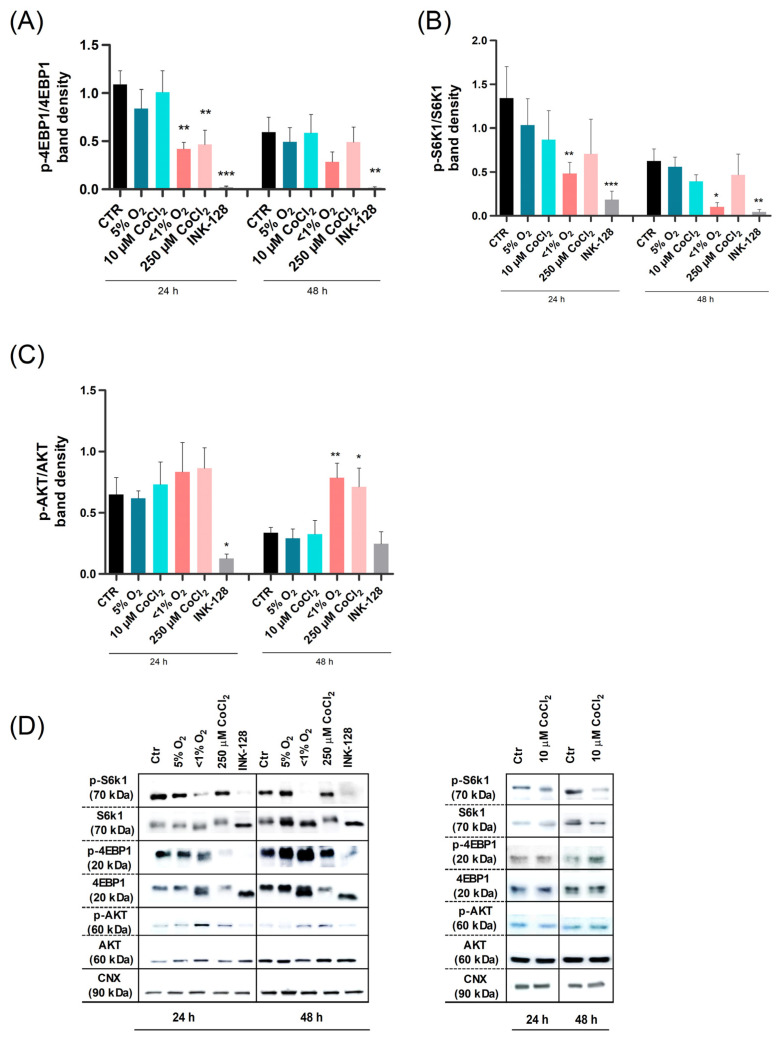
Effect of hypoxia on UC-MSCs is linked to the mTOR pathway. UC-MSCs underwent control (21% O_2_ and INK-128) and experimental conditions (5% O_2_, <1% O_2_, 10 μM CoCl_2_, and 250 μMCoCl_2_) for 24 and 48 h, after which protein analysis was carried out via Western blot. (**A**–**C**) Protein band quantification of two mTORC1 targets (p-4EBP1/4EBP1 and p-S6K1/S6K1) and a mTORC2 target (p-Akt/Akt), normalized to the loading control calnexin (CNX). The ratio between phosphorylated and total forms could be used to infer protein function. (**D**) Representative immunoblot of the total and of the respective phosphorylated form of the 4EBP1, S6K1, and Akt proteins. Results are represented as means with SEM of a minimum of 3 independent experiments. Statistical significance considered when * *p* < 0.05%, ** *p* < 0.01, and *** *p* < 0.001.

Following exposure to severe hypoxia (both physical and chemical), the UC-MSCs exhibited a significant decrease in cell culture proliferation. In order to test our initial hypothesis that these proliferative changes were caused by an mTOR signaling inhibition, we evaluated the expression levels of the phosphorylated and total forms of key mTORC1 (4EBP1 and S6k1) and mTORC2 (protein kinase B, AKT) downstream effectors (Figure 3).

The expression levels of p-4EBP1/4EBP1 and p-S6K1/S6K1 were shown to be similar between cells exposed to both 5% O_2_ and 10 µM CoCl_2_, with no statistical differences in relation to the control (Figure 3A,B). Inversely, while exposure to both <1% O_2_ and 250 μM CoCl_2_ (24 h) significantly decreased p-4EBP1/4EBP1 expression levels, a similar decrease in p-S6K1/S6K1 was only discerned in cells treated under <1% O_2_. Surprisingly, the expression of the mTORC2 survival-related protein Akt was increased in UC-MSCs exposed to severe hypoxia and to 250 µM CoCl_2_, with a significant increase after 48 h (Figure 3C). As expected, our internal control, INK-128, was able to promote an accentuated reduction in the active form of all mTORC1 and mTORC2 effectors evaluated in this study.

These results suggest that overall translation is affected in UC-MSCs under severe hypoxia through mTORC1 modulation, and that the same conditions strengthen UC-MSCs survival mechanisms via mTORC2 regulation.

## 3. Discussion

Quiescence is a physiologic state for many adult stem cells in their natural niche [23]. The traditional perspective that quiescence is a passive cellular state has been shifting in recent years, as we have progressed in understanding its biological significance in stem cell function. In physiological conditions, a low oxygen supply maintains the resident MSC pool in a reversible slowly cycling state that maintains tissue turnover as well as being responsible for preserving stemness and providing a source of cells ready to be activated for regenerative purposes [14]. Subtle changes in oxygen supply, however, trigger homing signals that mobilize these stem cells from their native niche. Thus, it is of utmost importance to understand the implications that oxygen tensions have in stem cell fate. Inconsistencies between reports are likely a result of the different strategies employed to mimic hypoxic O_2_ tension. Moderate hypoxia (3–5% O_2_) has been consistently shown to stimulate MSC proliferation [7,9]. However, different O_2_ levels can lead to distinct results in terms of MSC proliferation, differentiation capacity, and viability [11].

Nevertheless, CoCl_2_ has been extensively used as a hypoxia-mimicking agent for MSC lineages, as this compound is considerably more affordable than the costly hypoxia incubation systems and allows for longer periods of HIF stabilization [24]. Zeng et al. compared the effects of a range of CoCl_2_ concentrations (10 μM, 25 μM, 50 μM, and 100 μM) in UC-MSCs, and attested that this mimetic could inhibit MSC proliferation in a dose-dependent manner; however, the author still asserts that there are incongruities between physical hypoxia and CoCl_2_-induced hypoxia [25]. Therefore, whether CoCl_2_ is a reliable hypoxia substitute or not remains elusive. Ultimately, most studies either report CoCl_2_ as an unreliable hypoxic mimetic for MSCs—when this compound provokes a cellular response that differs from the effects of a specific O_2_ percentage—or fail to compare physical hypoxia to their CoCl_2_ condition, instead comparing their results with what was previously described in the literature [26,27].

Accordingly, in the present study, we proposed the characterization of a hypoxia chemical-inducer model for UC-MSCs, establishing a comparison between CoCl_2_ concentrations and a range of O_2_ tensions, at a cellular and molecular level. We assessed UC-MSCs under different oxygen tensions, modulated through different fashions, to determine differences in an arrested or actively proliferating profile; in addition, we identified the metabolic and biosynthetic pattern—by means of mTOR activity—from each individual experimental condition. We showed that a sharp decrease in oxygen availability (<1%) potentiated a glycolytic metabolism and cell quiescence, characteristic of many adult stem cells in their native niche [2].

Oxygen serves as a terminal electron acceptor for energy production in mitochondrial electron transport chains. Considering its limited availability under hypoxic conditions and the subsequent increase in HIF1α mobilization, a shift to glycolysis is induced [28]. The molecular triggers for this include the glucose transporter GLUT1, pyruvate dehydrogenase kinase 1 (PDK1), and LDHA [29]. While GLUT1 promotes the uptake of glucose, PDK1, by inhibiting pyruvate dehydrogenase [30], it suppresses the flow of pyruvate to the TCA cycle; LDHA, in turn, facilitates anaerobic respiration by converting pyruvate into lactate [31]. However, as the present study attests, different O_2_ levels prompt different metabolic pathways. With severe hypoxia, the ensuing exclusive dependency on anaerobic mechanisms, as a means of producing ATP, is thought to safeguard the cell against oxidative stress, DNA damage, and cellular ageing. Conversely, in less severe hypoxic environments (under 5% O_2_), the level of ROS increases and the cells are stimulated to proliferate and differentiate [32]. Accordingly, to support the elevated biosynthetic requirements of highly proliferating cells, cells exhibit a hypermetabolic state relying on both oxidative phosphorylation as well as glycolysis.

The present study also found a disparity in both OCR and COX IV levels between cells under physical (low O_2_) and chemical (CoCl_2_) hypoxia: COX IV was slightly decreased under 10 µM CoCl_2_—which goes in hand with its OCR results—while it had a slight increase in cells that underwent 5% O_2_—which, in turn, could allude to a corresponding increase in OCR. The same could be inferred from our ECAR results, which, together with the LDHA data, could suggest an increase in glycolysis for cells under 5% O_2_.

The limiting criteria in using CoCl_2_ as a mimic of moderate hypoxia would come from its different metabolic effects possibly induced by HIF-independent responses triggered by physical hypoxia, but not produced by chemical induction, namely through the c-Myc signaling pathway. C-Myc is a transcription factor that promotes both mitochondrial metabolism and glycolytic gene expression and is activated by mTOR signaling [33]. Alternatively, some authors argue that different oxygen levels and CoCl_2_ concentrations prompt different cellular responses associated with the heterogeneity of the efficiency in the hydroxylation of HIF-1α and HIF-2α, according to the severity of the hypoxic setting and CoCl_2_ concentration used. HIF-1α levels increase as O_2_ levels lower to severe hypoxia, and HIF-2α levels increase with moderate hypoxia [34]. In our hypoproliferative conditions, the inhibition of 4EBP1 and S6K1, associated with RNA translation and protein synthesis suppression, was consistent with a decrease in cell growth and proliferation. Likewise, Akt protein analysis showed that severe hypoxia displayed an increase in Akt phosphorylation under <1% O_2_, further supporting that a precursory quiescent state drives glycolysis and increases UC-MSC survival strategies. Finally, these results also showed that severe hypoxia acts on translational/biosynthetic mechanisms activated by mTORC1 and on metabolic/survival pathways activated by mTORC2.

**Figure 4:**
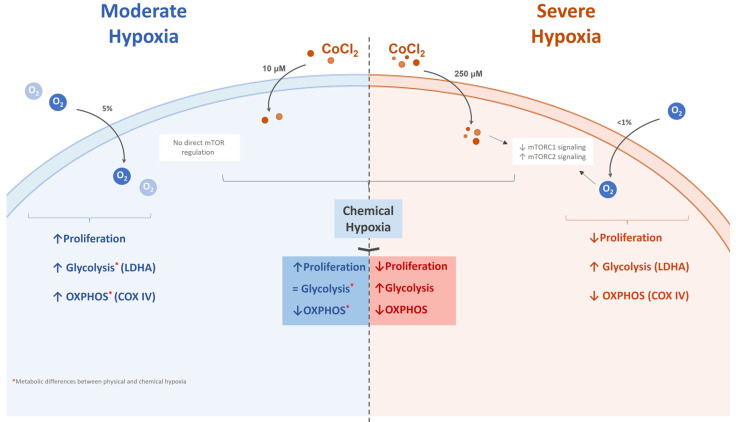
Effects of the different hypoxia conditions on UC-MSC behavior described in this work.

In summary, we determined the hypoxic condition that prompts a quiescence-like state in UC-MSCs that seems similar to what has been described in embryonic stem cells [12,35]. This is characterized by a reversible mTOR-mediated arrest in proliferation, which we also previously showed to imply a hypometabolic state in embryonic stem cells [18], similarly to what, following some preliminary work involving also another mTOR inhibitor (rapamycin, data not shown) we described here for UC-MSC. However, more work is needed to fully validate this approach as well as its possible relevance in MSC storage or practical use. We characterized this cellular state at a proliferative, metabolic, and mTOR level, comparing gas-controlled hypoxia with its CoCl_2_ counterpart. The differences described in the several hypoxia systems used in this work (moderate/severe; gas/chemical) are summarized in Figure 4.

## 4. Materials and Methods

### 4.1. Cell Culture

The proposal was approved by the Faculty of Medicine of the University of Coimbra’s Ethics Committee and followed appropriate informed consent and data protection guidelines at the University of Coimbra (process approval #CE-131/2020). Human umbilical cord-mesenchymal stem cells (UC-MSCs) were obtained after written informed consent, in compliance with ethical regulations and according to the approved ethical protocol and the data protection guidelines that were in place. UC-MSCs were plated at a density of 4 × 10^3^ cells/cm^2^ in α-minimum essential medium supplemented with 10% (*v/v*) fetal bovine serum, 100 U/mL of penicillin, 100 mg/mL streptomycin, and 1% amphotericin B (all from Thermo-Fisher Scientific, Waltham, MA, USA). Cells were grown in a humidified incubator at 37 °C with 5% CO_2_. Passages between 1 and 3 were used for the ensuing experiments.

### 4.2. Hypoxic Treatment and Experimental Design

UC-MSCs were incubated under different O2 concentrations for 24 and 48 h: 5% O_2_ in a humidified incubator (5% CO_2_, 90% N2, 37 °C); 1% O_2_ in a hypoxia chamber (STEMCELL Technologies Inc., Cambridge, MA, USA) (5% CO_2_, 94% N2, 37 °C); <1% O_2_ in a BD GasPak EZ anaerobe gas-generating pouch system (Becton, Dickinson and Company, Sparks, MD, USA) at 37 °C [36]. For chemical hypoxia induction, culture media was exchanged by medium containing the hypoxia-mimicking agent cobalt (II) chloride hexahydrate (CoCl_2_.6H_2_O; Sigma-Aldrich, Burlington, MA, USA) [24] at several concentrations (10 µM, 50 µM, 100 µM, 200 µM, and 250 µM), after which the cells were incubated for 24 and 48 h (21% O_2_, 5% CO_2_, 37 °C). To induce a paused-like state, and as a control condition for mTOR dual inhibition, cells were treated with the mTOR pharmacological inhibitor INK-128 (100 nM, MedChem Express, Monmouth Junction, NJ, USA).

### 4.3. Live Cell Imaging, Cell Growth and Cell Viability

Representative images of UC-MSCs were obtained from randomly selected plate fields with a phase-contrast microscope (Leica DMI3000B) and a Leica DFC425C camera. To evaluate growth rate and cell viability, cells dissociated with trypsin were stained with trypan blue (1:1; Sigma-Aldrich) and counted in a phase-contrast microscope using a Bright-Line™ hemacytometer (Sigma-Aldrich). The growth rate was calculated using the following mathematical formula: Growth rate = (ln(x + 24 h) − ln(x))/(24 h), where x stands for the total number of cells at one-time point and x + 24 h stands for the total number of cells counted 24 h after x. Cell viability was calculated by dividing the number of dead cells by the total number of cells.

### 4.4. Flow Cytometry

Flow cytometry was performed to identify apoptotic and necrotic profiles. A total of 20 × 10^3^ cells per condition were gated and evaluated using a FACScalibur flow cytometer (BD Biosciences, Franklin Lakes, NJ, USA) and a Cell Quest Pro Acquisition software (version 5.1, BD Biosciences). Using the fluorescent probes Annexin V (Immunostep, Salamanca, Spain) and propidium iodide (PI), four populations were identified: live cells (negative for both Annexin V and PI); early apoptotic cells (positive for Annexin V and negative for PI); late apoptotic cells (positive for both Annexin V and PI); and dead cells (only positive for PI). Cells were detached by trypsinization and centrifuged (550× *g*, 6 min), 106/mL cells were resuspended in Annexin V binding buffer (10 mM Hepes/NaOH (pH 7.4), 140 mM NaCl, 2.5 mM CaCl_2_), and they were incubated with Annexin V (50 µL/mL) and PI (2.5 µg/mL) for 15 min at RT, protected from light. Cells incubated overnight with 300 µM hydrogen peroxide (H_2_O_2_; Sigma-Aldrich) were used as a positive control for cell death.

### 4.5. Western Blotting

Samples were collected by lysing with RIPA lysis buffer (Sigma-Aldrich), supplemented with phenylmethanesulfonyl fluoride (PMSF, Sigma-Aldrich), 2 × Halt phosphatase inhibitor cocktail (Themo Fisher Scientific, Waltham, MA, USA), and CLAP protease inhibitor cocktail (Sigma-Aldrich). Proteins were quantified using the Pierce™ BCA Protein Assay Kit (ThermoFisher Scientific) according to the manufacturer’s instructions. Samples were then denatured by dilution with Laemmli sample buffer (Bio-Rad, Hercules, CA, USA)/β-mercaptoethanol and by heating at 70 °C for 10 min. Equivalent amounts of total protein (15 µg) were separated by electrophoresis in 7% or 14% Acrylamide Tris-HCl gels (Bio-Rad) and subsequently transferred into Immuno-Blot^®^ PVDF membranes (Bio-Rad). After blocking for 1 h with 5% bovine serum albumin in TBS-T (137 mM NaCl, 19 mM Tris-base, 0.1% Tween-20, pH = 7.6), membranes were incubated overnight at 4 °C with the primary antibodies specific to 4EBP1 (Cat. No:#9644), p-4EBP1 (Thr37/46) (Cat. No:#9459), S6K1 (Cat. No:#2708), p-S6K1 (T389) (Cat. No:#4691), AKT (Cat. No:#4691), p-AKT (Ser473) (Cat. No:#4058), LDHA (Cat. No:#3582), p-LDHA (Tyr10) (Cat. No:#8176), and COX IV(Cat. No: #11967), all from Cell Signalling Technology (Danvers, MA, USA), and used at a dilution of (1:1000). After washing, the membranes were incubated at RT for 1 h with the respective peroxidase-conjugated secondary antibodies (goat anti-mouse) or goat anti-rabbit (Bio-rad) (1:2000)). Protein detection was carried out using Clarity Western ECL Substrate (Bio-Rad), in an ImageQuant LAS 500 system (GE Healthcare, Uppsala, Sweden). The results were normalized with Calnexin (SICGEN, Cantanhede, Portugal), and the ImageJ software (v1.53e; NIH, Bethesda, MD, USA) was used to quantify protein band densities.

### 4.6. Live Cell Metabolic Analysis

Glycolytic function and mitochondrial bioenergetics were studied by assessing extracellular acidification rates (ECAR) and oxygen consumption rates (OCR), respectively, using a Seahorse XF24 Analyzer (Agilent Technologies, Santa Clara, CA, USA) [37]. The Seahorse XF24 Analyzer is a sensitive, high-throughput instrument that makes real-time measurements of medium acidification and respiration rates. In this assay, 50 × 10^3^ cells were plated in a Seahorse 24-well culture microplate (Seahorse Bioscience)—in culture medium (control) or supplemented with 10 µM CoCl_2_, 250 µM CoCl_2_—and allowed to adhere overnight. One hour prior to the assay, the culture medium was replaced by the appropriate XF Assay Medium: to measure glycolytic function, the medium was supplemented with L-glutamine (2 mM) and adjusted at pH = 7.4; to measure mitochondrial function, the medium was supplemented with 4.5 g/L glucose, 2 mM pyruvate, and 2 mM L-glutamine and adjusted at pH = 7.4. Cells were incubated at 37 °C for 1 h in the absence of CO_2_. OCR and ECAR were monitored in real-time, according to the manufacturer’s standard protocol. To measure glycolytic function, glucose (10 mM), oligomycin (1 μM), and 2-deoxyglucose (100 mM) were sequentially injected after measurements 3, 6, and 9, respectively. To assess mitochondrial function, oligomycin (1 μM), FCCP (1.25 μM), and rotenone + antimycin A (1 μM each) were injected after measurements 3, 6, and 9, respectively. As a blank control, ECAR and OCR were measured in a plate devoid of cells. At the end of the assay, the cells were displaced by trypsinization and counted to normalize the Seahorse raw data. Glycolysis, glycolytic capacity and reserve, basal respiration, maximum O_2_ consumption, and estimated ATP turnover were calculated using the second time-point measurement after the compound injection (measurements 2, 5, 8, and 11). For the OCR assay, parameters were measured using the formulas: basal respiration (measurement 3); ATP-linked respiration (basal respiration—measurement 6); maximal respiration (measurement 9—measurement 12); spare capacity (maximal respiration—basal respiration). For the ECAR assay, parameters were measured using the following formulas: glycolysis (measurement 6—measurement 3); glycolytic capacity (measurement 9—measurement 3); glycolytic reserve (measurement 9—measurement 6); non-glycolytic acidification (measurement 3).

### 4.7. Statistical Analysis

Data analysis was performed by using the GraphPad Prism 9.0 program (GraphPad Software Inc., San Diego, CA, USA). All data are expressed as mean ± standard errors of the means (SEM). Multiple comparisons were performed using one-way analysis of variance (ANOVA) followed by the Bonferroni multiple comparison post hoc test and the t-test was conducted in proliferation studies to compare the two groups. Statistical significance was considered at * *p* < 0.05%, ** *p* < 0.01, and *** *p* < 0.001.

## 5. Conclusions

We were able to determine the direct effects of severe and moderate hypoxia on UC-MSCs and confirm that severe hypoxia is, indeed, involved in keeping the cells in a quiescent state that drives glycolysis. This state is characterized by a decrease in the activity of mTORC1 effectors that regulate proliferation and global biosynthesis, and due to an increase in mTORC2 survival proteins, is also implicated in glycolytic metabolism. Finally, we were able to perform a comparison between O_2_ levels and a range of CoCl_2_ concentrations, based on their cellular and molecular similarities. Nevertheless, as our results suggest, CoCl_2_ prompts a different metabolic response, distinct from that of cells exposed to low oxygen tension in proper incubation systems. Additionally, the usage of CoCl_2_ does not account for HIF-independent mechanisms, so by using this compound as a substitute for “natural” hypoxia, these disparities should be considered for different research prospects.
